# Autotoxic Ginsenosides in the Rhizosphere Contribute to the Replant Failure of *Panax notoginseng*

**DOI:** 10.1371/journal.pone.0118555

**Published:** 2015-02-19

**Authors:** Min Yang, Xiaodan Zhang, Yanguo Xu, Xinyue Mei, Bingbing Jiang, Jingjing Liao, Zhaobo Yin, Jianfen Zheng, Zhi Zhao, Liming Fan, Xiahong He, Youyong Zhu, Shusheng Zhu

**Affiliations:** Key Laboratory of Agro-Biodiversity and Pest Management of Education Ministry of China, Yunnan Agricultural University, Kunming, China; Northwest A&F University, CHINA

## Abstract

**Background and Aims:**

Sanqi ginseng (*Panax notoginseng*) growth is often hampered by replant failure. In this study, we aimed to examine the role of autotoxicity in Sanqi replant failures and assess the role of ginsenosides in autotoxicity.

**Methods:**

The autotoxicities were measured using seedling emergence bioassays and root cell vigor staining. The ginsenosides in the roots, soils, and root exudates were identified with HPLC-MS.

**Results:**

The seedling emergence and survival rate decreased significantly with the continuous number of planting years from one to three years. The root exudates, root extracts, and extracts from consecutively cultivated soils also showed significant autotoxicity against seedling emergence and growth. Ginsenosides, including R_1_, Rg_1_, Re, Rb_1_, Rb_3_, Rg_2_, and Rd, were identified in the roots and consecutively cultivated soil. The ginsenosides, Rg_1_, Re, Rg_2_, and Rd, were identified in the root exudates. Furthermore, the ginsenosides, R_1_, Rg_1_, Re, Rg_2_, and Rd, caused autotoxicity against seedling emergence and growth and root cell vigor at a concentration of 1.0 µg/mL.

**Conclusion:**

Our results demonstrated that autotoxicity results in replant failure of Sanqi ginseng. While Sanqi ginseng consecutively cultivated, some ginsenosides can accumulate in rhizosphere soils through root exudates or root decomposition, which impedes seedling emergence and growth.

## Introduction

Sanqi ginseng [*Panax notoginseng* (Burk.) F. H. Chen], a member of the Araliaceae family, is one of the most important herbal medicines in China and is in high demand [1]. In China, the commercial cultivation of Sanqi ginseng has been practiced for more than 200 years. Because ginsenosides have significant effects against cancer and cardiovascular disease [2], requirement for these compounds have increased rapidly. Recently, more than 4 million hectares of Sanqi ginseng have been sown annually in China. However, Sanqi ginseng plants are often hampered by replant failure, which results in yield reductions and other difficulties when reestablishing plants in cultivated fields due to low seed germination, poor seedling growth, and severe disease [3]. Generally, rotation is the preferred method for avoiding crop replant failure. However, more than 30 years of rotation are necessary for successful Sanqi ginseng replanting [3]. Therefore, one common farming practice is to avoid building Sanqi ginseng gardens in the same area. Many factors potentially contribute to this problem, including the deterioration of soil physicochemical characteristics, nutrient imbalance, soil-borne diseases, and autotoxicity [4–5]. In the last three decades, researchers and farmers have hypothesized that Sanqi ginseng replant failure results from the accumulation of pathogens in the soil. Many types of soil-borne pathogens that cause diseases in Sanqi ginseng have been identified, including fungi, Oomycetes, bacteria, nematodes, and viruses [6–9]. However, Sanqi ginseng cannot be successfully cultivated again on the same land even after sterilizing the soil by using fungicides or fumigation [10]. In addition, many tested fertilizer application and soil modification practices have yielded unsatisfactory results [11].

Recently, it was hypothesized that the secondary metabolites released by plant residues and root exudates that accumulated in the soil could be autotoxic to *P*. *notoginseng* during replanting. Autotoxicity, which is defined as a deleterious allelopathic effect among individuals of the same species, has been documented in various crops [12–13]. For many medicinal plants, such as *Rehmannia glutinosa*, *Pseudostellaria heterophylla* and *Salvia miltiorrhiza*, autotoxicity results in significantly reduced yields and quality [14–17]. The autotoxicities in ginseng (*P*. *ginseng*) and American ginseng (*P*. *quinquefolius*), which belong to the same genus as Sanqi ginseng (*P*. *notoginseng*), were recently reported as one possible factor that contributes to replant failure [18–24]. Some phenolic acids in the root exudates and rhizosphere soils of American ginseng have been identified as potential autotoxic compounds [20]. However, phenolic acids have frequently been identified in other crops as putative allelopathic substances and have been identified in uncultured soil [20, 25]. Thus, we speculated that other special autotoxic compounds might result in Sanqi ginseng replant failure.

Ginsenosides are the primary biologically active compounds that are produced by Sanqi ginseng plants. At least 20 different ginsenosides occur in Sanqi ginseng, which account for more than 6% of the plant's dry biomass [26]. Ginsenosides can be released into the rhizosphere soil as root exudates, by leaching and volatilization, or from the decomposition of plant residues by Sanqi [1] and American ginseng [27–28]. Although many studies have examined the pharmacological properties of ginsenosides, little is known regarding their ecological roles in the soil following their release from Sanqi plants [2]. However, small concentrations of ginsenoside may play a role in ginseng replant failure. Some studies have indicated that ginsenosides can act as allelopathic stimulators for the growth of important ginseng pathogens, such as *Pythium irregulare*, *Cylindrocarpon destructans*, *Phytophthora cactorum*, and *Fusarium solani* [27–30]. However, limited data are available regarding the autotoxicity effects of ginsenosides on Sanqi ginseng.

In this study, we aim to (i) confirm the role of autotoxicity in the replant failure of Sanqi ginseng, (ii) identify and quantify ginsenosides in the root exudates and soil plow layer of Sanqi ginseng fields that have been consecutively cultivated for one to three years, and (iii) detect potentially autotoxic ginsenosides by using seed germination and root vigor staining methods.

## Materials and Methods

The field experiment was carried out in Experimental Station of Yunnan Agricultural University. These field studies were authorized by Yunnan Agricultural University, Yunnan, China. No specific permissions were required in these fields. We confirmed that the field experiment did not involve endangered or protected species.

### Plant growth conditions and root exudate collection

Healthy seeds from Sanqi ginseng were collected from mature plants that had been cultivated for three years from a commercial farm located in Yanshan County in Yunnan, China (104.32°E, 23.63°N, 1560 m Alt.). The seeds were surface-sterilized with 1% sodium hypochlorite for 5 min and washed three times with sterile water. The surface-sterilized seeds were transferred to the wells of seedling-raising plates (50-well plates, 5.0 cm ×5.0 cm × 8.0 cm per well). One seed was sown in each well, which contained 100 mL of soil (5 parts zeolite, 3 parts soil, and 2 parts organic fertilizer; pH 6.5; 14% organic matter; EC: 0.224 Ms cm^-1^; and nutrient contents of 82.8 mg/kg available N, 31.2 mg/kg P, and 473 mg/kg K). The plates were placed in a Sanqi greenhouse located at the agricultural experiment station of Yunnan Agricultural University, Xundian county, Kunming, P. R. China (103.13°E, 25.67°N, 1880 m Alt.). Sanqi ginseng is an ombrophyte plant; therefore, the greenhouse was shaded with a polyethylene net that allowed 10% light transmission to imitate the natural conditions for Sanqi ginseng growth. The plants were initially irrigated once with 1/2 strength Hoagland's solution [31], and then twice a week with fresh water over the course of the experiment.

After three months of growth, the 8–10 cm high seedlings were uprooted from the substrates and the roots were gently washed with sterilized water to remove any adhered soil. Next, 60 seedlings were transplanted into plastic containers (27.0 cm ×20.0 cm × 11.5 cm), which contain 3.0 L of sterilized and double distilled water, to collect root exudates. All of the seedlings were cultured at 25°C with a 12 h light/12 h dark photoperiod. The solution in each container was continuously aerated (3.0 L min^-1^) using air pumps with two small air filters. After seven days of incubation, 12.0 L of root exudate solution was collected from 240 seedlings and filtered through a 0.22 μm nylon membrane filter (JTSF0311, Tianjin Jinteng Experiment Equipment Co. Ltd.). Next, 8.0 L of the root exudates was used for a hydroponics experiment. The remaining 4.0 L were concentrated and dried under reduced pressure (Rotavapor R-200, Büchi). Finally, the concentrate was dissolved in methanol and then passed through a 0.22 μm nylon membrane filter (JTSF0311, Tianjin Jinteng Experiment Equipment Co. Ltd.). The filtered solution was stored at 4°C for further experiments. The experiment was repeated three times.

### Soil and Sanqi ginseng roots collection

Soil samples were collected in October 2012 from a Sanqi ginseng field in Yanshan County (104.32°E, 23.63°N, 1560m Alt.), which is located in an optimal production area for Sanqi ginseng. 1Plow soil samples (20 cm depth) were collected during harvest from fields that were consecutively cultivated for one to three years. A soil sample was collected from an adjacent uncultivated field as a control. At each collection site, nine samples were randomly collected and pooled into three samples. The soil samples were sieved through a 40-mesh screen to remove plant debris before air-drying and stored at 4°C until further analysis. Healthy and fresh three-year-cultivated Sanqi ginseng roots were collected from the same field in 2012. Three sites were selected to collected roots. Nine samples were randomly collected and pooled into three samples at each collection site. The roots were carefully washed and air-dried and then ground into a powder using a blender (DF-20, Linda machinery co. ltd., China) and passed through a screen (mesh 100).

### Effects of continuously cultivated soils on Sanqi ginseng growth

To assess the replant failure of Sanqi ginseng, the above collected soil, including three consecutively cultivated soils from one to three years and one uncultivated soil, were used to test their effect on seedling emergence and growth. Soils were placed in 50-well seedling-raising plates. Next, the seeds were sown as described above. Each treatment contained three replicates, and each replicate contained three seedling-raising plates. All plates were arranged in the same greenhouse in a completely randomized block design. The seedling emergence rate was recorded when the emergence rate of the uncultivated soil control treatment exceeded 80%. The survival rate of the seedlings was surveyed after six months of seedling emergence. And then, all of the seedlings were harvested, and the height and fresh biomass of the seedlings were measured.

### Effect of root powder on Sanqi ginseng growth in the uncultivated soil

To investigate the effects of root residue on Sanqi ginseng growth, different concentrations of root powder were added to the uncultivated soil. Next, their effects on seed emergence and seedling growth were assessed. The root powder was mixed with uncultivated soil at concentrations of 0, 0.05, 0.10, 0.50, 1.00, 2.00, and 5.00 g L^-1^. All the soils were then distributed into 50-well seedling-raising plates. Each treatment contained three replicates, and each replicate contained three seedling- raising plates. Next, 50 surface-sterilized seeds were sown in each plate. All of the plates were arranged in the same greenhouse in a completely randomized block design. The seed emergence and seedling growth were measured as detailed described above.

### Effects of autotoxicity on Sanqi ginseng growth under hydroponic conditions

The autotoxicity of Sanqi ginseng was investigated by using hydroponics with or without activated charcoal (AC), as described by Asaduzzaman et al. [32]. Overall, four treatments were used in the experiment. Two treatments consisted of seedlings that were incubated in a container filled with 2.0 L of 1/10 strength Hoagland's solution with or without AC (4–8 mesh, Tianjin Fengchuan Chemical Reagent Science and Technology Co. Tianjin, China). The other two treatments involved the incubation of seedlings in a container filled with 1.0 L of 1/5 strength Hoagland's solution mixed with 1.0 L of root exudates with or without AC. Sixty seedlings were planted in each container (27.0 cm × 20.0 cm × 11.5 cm), and three containers were used for each treatment based on a randomized block design. The solution in each container was continuously aerated (3.0 L min^-1^) using air pumps with two small air filters. The AC treatments were mixed with 24 g of AC in each container according to Yu’s method [33]. The same aeration system was maintained for the solution without AC. During cultivation, the water levels of the solution containers were held constant by regularly adding deionized water to compensate for evaporation. The seedling survival rate was recorded after thirty days of incubation.

### Soil sample extraction

Air-dried soil samples (500 g) were extracted for 24 h with 1,500 ml MeOH:H_2_O (80:20) on an orbital shaker (ZHWY-111B, Shanghai ZHICHENG Analytical Instruments Manufacturing Co., Ltd) at 120 rpm at 25 ± 2°C. The extract was vacuum-filtered through a piece of No. 4 quantitative filter paper (Whatman, 9.0 cm diameter). The solutions from the three extractions were combined and evaporated to dryness under a vacuum at 40°C. The dry residue from the evaporation was redissolved in 100% MeOH to a concentration of 0.1 g mL^-1^ and then passed through a 0.22 μm nylon membrane filter (JTSF0311, Tianjin Jinteng Experiment Equipment Co. Ltd.). The filtered solution was stored at 4°C for further experiments. Three samples were extracted from each collection site.

### Extraction of the crude ginseng saponin fraction from root powder

One grams of root powder from three-year-cultivated Sanqi ginseng roots were poured into a 15 mL tube and ultrasonically extracted at a concentration of 1 g per 4 mL with MeOH:H_2_O (80:20) at 40°C for 40 min. Next, the tube was centrifuged for 5 min (12,000 g, 4°C). The extraction procedure was performed three times. The solutions from the three extractions were combined and evaporated to dryness under a vacuum at 40°C. The dry residue was redissolved in 100% MeOH to a concentration of 0.1 g mL^-1^ and passed through a 0.22 μm nylon membrane filter (JTSF0311, Tianjin Jinteng Experiment Equipment Co. Ltd.). The filtered solution was stored at 4°C for further experiments. Three samples were extracted from three-year-cultivated Sanqi ginseng roots.

### Autotoxicity bioassays

To identify the effects of potentially autotoxic compounds on Sanqi ginseng growth, bioassays were performed as described by Zhao et al. (2005) with some modifications [24]. Briefly, the root exudates, the extracts from the roots and soils that were consecutively cultivated for one to three years, and the eight individual ginsenosides (R_1_, Rg_1_, Re, Rf, Rb_1_, Rg_2_, Rg_3_, Rd, Rb_3_, and Rh_1_, purity≧98%, Guizhou Dida Biological Technology Co.) were each dissolved in a small volume of methanol and diluted in distilled water to a concentration of 1.0 mg L^-1^. In addition, distilled water containing the same concentration of methanol (1.0%) was used as a control. Sanqi ginseng seeds were surface-sterilized with 1% sodium hypochlorite for 5 min and then washed three times with sterilized water. Glass bottles (250 mL) containing 100 g of the coarse silica sand were sterilized at 160°C for 3 h. Ten seeds were placed in the silica sand in each bottle, and 10 mL of the respective treatment solution was then added. Each treatment was replicated four times using a randomized design, and the bioassays were repeated twice. The seeds were incubated in a programmable illuminated incubator with an L/D cycle of 12 h/12 h and a temperature cycle of 25°C/20°C (day/night). The emergence rate, root length, plant height, and fresh biomass of the seedlings were measured after the emergence rate of the control treatment exceeded 80%.

### HPLC-ESI-MS analysis

The ginsenosides were identified and quantified using an HP 1100 HPLC system fitted with a diode array detector (DAD) that was directly connected to a Bruker Esquire HCT Esquire 3000 electrospray ionization (ESI) ion trap mass spectrometer (Bruker Daltonik GmbH, Shanghai, China) in negative ion mode. The HPLC separations were performed using a DIONEX-C18 reverse-phase column (150mm × 4.6mm, 5μm). A multistep gradient was used for all separations with an initial injection volume of 10 μl and a flow rate of 1.0 mL min^-1^. The solvent system consisted of the following linear gradient of solvent A (acetonitrile) and solvent B (0.1% phosphoric acid in water): from 1% to 18% A for 0 to 20.0 min, from 18% to 40% A for 20.0 to 40.0 min, from 40% to 55% A for 40.0 to 45.0 min, holding at 55% A for 5 min, and from 55% to 18% A for 50.0 to 51.0 min. The column temperature was maintained at 30°C. Chromatograms were recorded at 203 nm, and the following ESI-MS conditions were used: nitrogen (N_2_) was used as a nebulizer gas (25 psi) and a drying gas (10 L min^-1^ N_2_ at 330°C). The capillary voltage was set at 4000 V, the capillary exit was set at 106 V, and the skimmer was set at 40 V. The spectra were recorded at a normal resolution (full width of 0.6 u at half-peak height) under ion charge control (ICC) conditions (100,000) with a mass range of 25 to 1200 m/z and a trap drive value of 36.8 V. The chromatographic data were recorded and processed using a Waters empower workstation. The ginsenosides in the samples were identified by comparing the results to authentic ginsenoside standards. The concentration of ginsenosides in samples was quantified using standard curves that showed the linear relationships between the peak areas and the concentrations. The ginsenoside contents of samples were calculated by the following formula:

$$\text{Content of ginsenoside in roots or soil }(\text{µg}/\text{g})\;=1000\;\times \;(\text{m }\times \text{ V})/{\text{W}}_{1};$$ 1

$$\text{Content of ginsenoside in root exudates }(\text{mg}/\text{L})\;=\;(\text{m }\times \text{ V})/{\text{W}}_{2}$$ 2

where m is the ginsenoside content of sample extract as determined from a standard curve (mg/mL); V is the methanol volume (mL) to dissolve the dry residue of sample extract; W_1_ is the weight of the sample (g); and W_2_ is the volume of root exudates (L).

### Root cell death detection by propidium iodide (PI) staining

Sanqi ginseng seedlings were carefully collected from the soil and washed three times with sterilized water. Next, a 100 mL aliquot of the ginsenoside (R_1_, Rg_1_, Re, Rb_1_, Rg_2_, Rd, Rb_3_, or Rh_1_) solution at a concentration of 1.0 μg mL^-1^ was added to each sterilized glass bottle (250 mL). The same concentration of methanol was added to the sterilized water for the control. Six seedlings were placed in each bottle, and each treatment was replicated four times in a completely randomized block design. The seedlings were incubated in a programmable illuminated incubator with an L/D cycle of 12 h/12 h and a temperature cycle of 25°C/20°C. After incubating for 24 h, fibrous roots from the Sanqi ginseng seedlings that were longer than 2 cm were excised with a sterile razor blade. Changes in the root cell vigor were monitored using a modified capillary root model, as described by Fan et al. [34]. Briefly, the capillary tube (1 mm external diameter) was bent into a U-shape, placed on a glass slide and overlain with a cover slip to form a chamber with one open side. The tip of each fibrous root was inserted into the open end of the chamber and stained with PI (5 μg mL^-1^), as described previously [35]. The staining was recorded using a confocal video camera attached to a compound microscope (excitation 488 nm and emission above 630 nm; Leica DM2000, Germany). The number of dead cells in the apical and subapical roots was counted. The experiment was repeated three times, and 20 roots were tested for each treatment.

### Statistical analysis

All data were subject to an analysis of variance using PASW Statistics 18 (SPSS Inc.). Each value was expressed as the mean and standard error (SE). The significant differences in seed emergence and seedling growth between the treatments and control were calculated using a one-way analysis of variance (ANOVA) followed by a Fisher’s least significant difference (LSD) test. Principal component analysis (PCA) was used to identify the significant features for seed emergence and seedling growth for all treatments.

## Results

### Effects of consecutively cultivated soil on seedling emergence and growth

**Fig 1:**
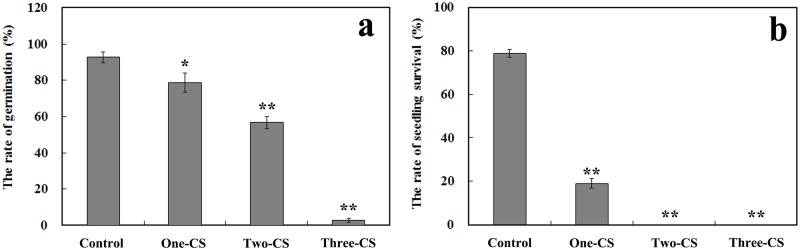
Seedling germination rate (a) and seedling survival rate (b) in continuously cultivated soil and uncultivated soil. Control represents the uncultivated soil, and One-CS, Two-CS, and Three-CS represent one, two, and three years of continuously cultivated soil. Error bars indicate the standard error (SE). Asterisks indicate statistically significant differences of treatment compared with control. *, *p* < 0.05; **, *p* < 0.01, LSD test.

The seeding emergence rate was significantly lower in the consecutively cultivated soils than in the uncultivated soil. In addition, as the continuous number of planting years increased, the emergence rate decreased significantly (Fig. 1 a). The emergence rate only reached 2.7% when the seeds were sown in soil that was continuously cultivated for three years. Six months after emergence, the seedling survival rate in the uncultivated soil was 78.7%. However, in the soils that were continuously cultivated for two and three years, the seedling survival rate was zero, and in the soil that was cultivated for one year, the seedling survival rate was 19.0% (Fig. 1 b).

### Effects of root exudates on seedling emergence and growth

**Fig 2:**
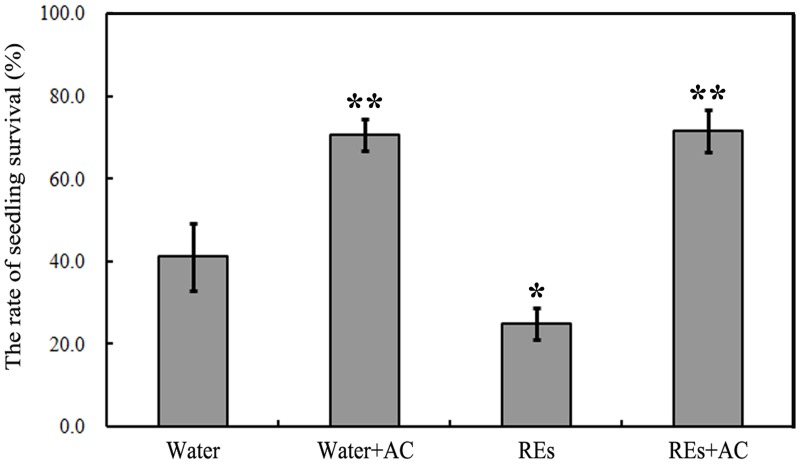
The effects of activated charcoal (AC) on the survival rate of Sanqi ginseng plants that were grown in water or root exudates (REs) with or without AC. The means and standard errors are shown. Asterisks indicate statistically significant difference of treatment compared with water control. *, *p* < 0.05; **, *p* < 0.01, LSD test.

The hydroponic experiments confirmed that the survival rate of the Sanqi ginseng plants significantly increased when grown in water or root exudates with added AC (Fig. 2). These results indicated that some abiotic factors might be present in the soil and root exudates that affect the growth of Sanqi ginseng.

### Effect of root residues on seed germination and seedling growth

The root powder of the Sanqi ginseng affected seed germination and seedling growth and exhibited a dose-dependent inhibition (Table 1). The average fresh biomass decreased significantly (*p* = 0.05 at 0.5 g L^-1^) in response to root powder treatments. Relative to the fresh biomass, the decrease in the plant height was lower, with significant effects observed when 1.0 g L^-1^ of root powder was added (*p* = 0.05). The decreases in the seedling emergence and survival rates were the lowest, with significant effects observed when 2.0 g L^-1^ of root powder was added in the soil (*p* = 0.05). These data indicated that there are potential inhibitors in the roots that affect the emergence and growth of Sanqi ginseng.

**Table 1:** Effects of root residue from Sanqi ginseng plants on seed emergence and seedling growth.

Concentration (g/L)	Seed emergence rate ± SE (%)^×^	Seedling survival rate ± SE (%)	Plant height ± SE (cm)	Fresh biomass ± SE (g)
**0.00**	90.67±3.71	78.33±2.60	9.08±0.18	1.57±0.07
**0.05**	92.00±2.00	70.67±3.71	8.71±0.18	1.51±0.06
**0.10**	92.67±0.67	68.67±4.81	8.93±0.20	1.67±0.09
**0.50**	85.33±2.40	63.33±5.33	8.55±0.16	1.42±0.07
**1.00**	83.33±1.76	62.67±1.76	8.27±0.19	1.39±0.12
**2.00**	71.33±4.81**	52.67±4.67**	8.12±0.21	1.38±0.08
**5.00**	66.67±1.76**	44.00±7.21**	7.89±0.21**	1.36±0.05*

^×^Means and standard errors (SE) are shown. Values shown here are the LSD statistic with a *p*-value < 0.05 marked as * and < 0.01 marked as **.

### Identification of ginsenosides from roots, soils and root exudates

**Fig 3:**
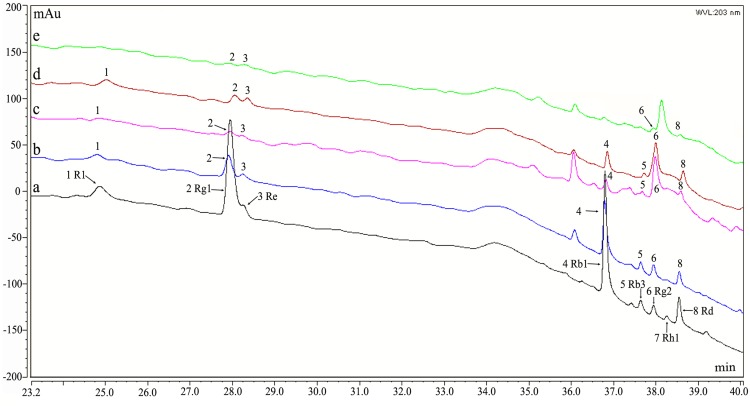
HPLC chromatograms of the Sanqi ginseng root extracts (a), three-year continuously cultivated soil (b), two-year continuously cultivated soil (c), one-year cultivated soil (d), and root exudates (e). Ginsenosides were detected in the samples in the order of appearance in the eluent: R_1_ (1), Rg_1_ (2), Re (3), Rb_1_ (4), Rb_3_ (5), Rg_2_ (6), Rh_1_ (7), and Rd (8).

Previous reports demonstrated that HPLC and MS data collected in negative ion mode are useful for identifying ginsenosides [36–37]. During root extraction, the R_1_, Rg_1_, Re, Rf, Rb_1_, Rb_3_, Rg_2_, Rh_1_, and Rd ginsenosides were identified according to their retention time (*t*_R_) and the characteristics of the deprotonated molecule ([M-H]^-^) relative to the authentic standards (Fig. 3, Table 2, S1 Dataset). The ginsenoside contents in the roots reached 6.54%. Eight ginsenosides, including R_1_, Rg_1_, Re, Rf, Rb_1_, Rb_3_, Rg_2_, and Rd, were recorded in the soil extracts from the plow layer of the continuously cultivated soils, for all three years (Table 2, S1 Dataset). The total ginsenoside contents in the different soil samples varied from 2.04 to 5.87 μg g^-1^ of the dry soil weight (Table 3, S2 Dataset). Thus, most of the ginsenosides identified in the Sanqi ginseng roots were also found in the soils that were previously cultivated with Sanqi ginseng. Furthermore, the Rg_1_, Re, Rg_2_, and Rd ginsenosides were identified in the root exudates using HPLC-MS (Table 2; S1 Dataset).

**Table 2:** Ginsenosides identified by HPLC-ESI-MS in the root tissues, soil extracts and root exudates of Sanqi ginseng.

Peak No.	Authentic ginsenoside standards				Primary ion fragments (m/z) in the samples with their corresponding retention times				
		*t*_R_ (min)	*M*_*W*_ (Da)	Primary ion fragments (m/z)	KG-3 *	One-CS	Two-CS	Three-CS	REs
**1**	R1	25.299	933	932 [M-H]^-^, 992 [M+AcOH-H]^-^	932, 992	932, 991.9	931.9, 992	931.9, 992	—
**2**	Rg1	28.077	801	800 [M-H]^-^, 859.9 [M+AcOH-H]^-^	859.9	859.9	859.9	800, 860	859.9
**3**	Re	28.707	947	946 [M-H]^-^, 1006 [M+AcOH-H]^-^	945.8, 1005.9	946.2, 1006	946.9, 1006	946.9, 1006	946.8, 1006.2
**4**	Rb1	36.872	1109	553.7 [M-2H]^2-^, 583.6 [M-H+OAc]^2-^, 799.9 [M-2Glc-CO_2_+OAc]^-^, 8859.9 [M-2Glc-CO_2_+OAc+AcOH]^-^, 1107.9 [M-H]^-^	553.7, 583.7, 860.0, 1108.7	553.7, 584.0, 800.0, 859.9, 1108.6	553.5, 583.7, 799.9, 860.0, 1108.3	553.7, 583.9, 799.9, 860.0, 1108.2	—
**5**	Rb3	37.713	1078	568.5 [M-H+AcOH]^2-^, 1077.9[M-H]^-^	568.5, 1078.3	568.8, 1078.1	568.8,1078.0	568.8, 1078.1	—
**6**	Rg2	38.093	784	783.9[M-H]^-^, 843.9[M+AcOH-H]^-^	783.8, 843.9	783.9, 843.9	784.9, 844	783.9, 844	844
**7**	Rh1	38.676	638.87	638.8[M-H]^-^, 697.8[M+AcOH-H]^-^	697.8	—	—	—	—
**8**	Rd	38.633	947	946[M-H]^-^, 1006[M+AcOH-H]^-^	945.9, 1006	946, 1006	946, 1005.9	946.1, 1007	945.9, 1006

*KG-3: Crude ginsenosides extracted from the three-year-old roots of Sanqi ginseng; One-CS, Two-CS, and Three-CS represent one, two, and three years of continuously cultivated soils, respectively. REs represent the root exudates of the Sanqi ginseng.

**Table 3:** Ginsenoside concentrations in the root tissues, soil extracts and root exudates of the Sanqi ginseng.

Peak No.	Ginsenosides	The calibration curve	Concentration (μg g^-1^) ± SE*				
			KG-3#	One-CS	Two-CS	Three-CS	REs
**1**	R1	y = 3592.5x—10.348, R² = 0.9990	4756.5±588.4	0.33±0.07	0.50±0.02	0.84±0.36	-
**2**	Rg1	y = 3406.9x—60.232, R² = 0.9997	26013.2±1177.0	0.66±0.04	1.14±0.02	1.71±0.71	0.28±0.02
**3**	Re	y = 3294.2x—7.0229, R² = 0.9994	2523.1±42.6	0.30±0.06	0.60±0.10	0.70±0.10	0.18±0.04
**4**	Rb1	y = 2523x- 26.227, R² = 0.9995	23603.6±405.7	0.24±0.10	0.69±0.35	0.57±0.01	-
**5**	Rb3	Y = 2819.2x—15.372, R² = 0.9992	859.6±61.9	0.14±0.02	0.23±0.02	0.25±0.01	-
**6**	Rg2	Y = 2183.7x—17.923, R² = 0.9991	1595.9±94.2	0.12±0.01	0.35±0.02	0.60±0.05	0.10 ±0.03
**7**	Rh1	y = 4636x + 2.8074, R² = 0.9992	874.8±82.9	-	-	-	-
**8**	Rd	y = 3175.4x-6.3896, R² = 0.9995	5170.8±493.3	0.25±0.01	0.65±0.11	1.20±0.06	0.22±0.03
Total			65397.5	2.04	4.16	5.87	0.78

*Means and standard errors (SE) are shown.

^#^KG-3: Crude ginsenosides extracted from the three-year-old roots of Sanqi ginseng; One-CS, Two-CS, and Three-CS represent one, two, and three years of continuously cultivated soils, respectively. REs represent the root exudates of the Sanqi ginseng.

-: undetected.

### Autotoxicity

The extracts from the soils that were consecutively cultivated for one to three years, the root exudates, and the crude ginsenosides significantly inhibited the seed germination and growth of Sanqi ginseng at a concentration of 1.0 μg mL^-1^ (Table 4). The seed germination, plant height, radicle length, and fresh biomasses of the seedlings were significantly reduced by 53.3~93.3%, 68.9~77.9%, 49.1~64.7%, and 68.4~73.7%, respectively. These data clearly demonstrated that potent inhibitors were present in the consecutively cultivated soils, root exudates, and crude ginsenosides. Interestingly, ginsenosides could contribute to the autotoxicity of Sanqi ginseng.

**Table 4:** Autotoxicities of the soil extracts, root extracts, root exudates, and ginsenosides on the seedling germination and growth of Sanqi ginseng.

Treatments ^#^	Seed germination rate (%) ± SE ^×^	Height (cm) ± SE	Root length (cm) ± SE	Fresh biomass (g) ± SE
**Control**	75.00±6.45	8.42±0.18	1.16±0.08	0.19±0.013
**Uncultured soil**	72.50±4.79	5.81±0.19	1.08±0.05	0.12±0.008
**CR**	17.50±4.80**	2.32±0.10**	0.43±0.03**	0.06±0.006**
**One-CS**	22.50±4.19**	2.21±0.19**	0.56±0.07**	0.07±0.006**
**Two-CS**	17.50±2.50**	1.86±0.31**	0.47±0.09**	0.05±0.005**
**Three-CS**	5.00±2.89**	2.32±0.19**	0.41±0.06**	0.06±0.008**
**Res**	35.00±7.1**	2.62±0.12**	0.59±0.10**	0.06±0.004**
**R1**	40.00±4.08**	3.29±0.34**	0.67±0.06**	0.08±0.010**
**Rg1**	50.00±9.13*	2.25±0.40**	0.44±0.09**	0.04±0.012**
**Re**	45.00±2.89**	1.87±0.15**	0.49±0.07**	0.05±0.009**
**Rf**	52.50±4.79*	5.23±0.60	0.83±0.08*	0.12±0.009
**Rb1**	57.50±4.79*	4.65±0.46*	0.75±0.08*	0.11±0.012
**Rg2**	35.00±9.57**	1.71±0.20**	0.47±0.06**	0.05±0.004**
**Rg3**	67.50±7.50	5.01±0.13	0.78±0.04*	0.09±0.006**
**Rd**	37.50±4.79**	2.96±0.17**	0.60±0.07**	0.07±0.010**
**Rb3**	80.0±8.16	4.97±0.43	0.96±0.10	0.14±0.007
**Rh1**	55.00±9.57*	5.06±0.29	1.03±0.08	0.13±0.005

^#^ One-CS, Two-CS, and Three-CS represent the soil samples from the fields with one, two, and three years of continuous Sanqi ginseng cultivation. CR represents the crude ginsenosides that were extracted from the three-year Sanqi ginseng roots. REs represent the root exudates of the Sanqi ginseng.

^×^ Means and standard errors (SE) are shown. Values shown here are the *F* statistic with a *p*-value < 0.05 marked as * and < 0.01 marked as **.

Based on the identification and availability of the ginsenosides, ten individual compounds were selected for examination of their potential autotoxic effects on Sanqi ginseng seedling germination and growth. Among the ten ginsenosides examined here, six ginsenosides significantly inhibited seedling germination and growth (Table 4). Specifically, the seedling germination, plant height, radicle length and fresh seedling biomass were significantly reduced by 33.3~53.3%, 60.9~79.7%, 42.2~62.1% and 57.9~78.9%, respectively, when they were treated with the R_1_, Rg_1_, Re, Rb_1_, Rg_2_, and Rd ginsenosides at 1.0 μg mL^-1^ (Table 4). These results clearly demonstrated that the ginsenosides inhibited seed germination and seedling growth at the soil relevant concentrations.

**Fig 4:**
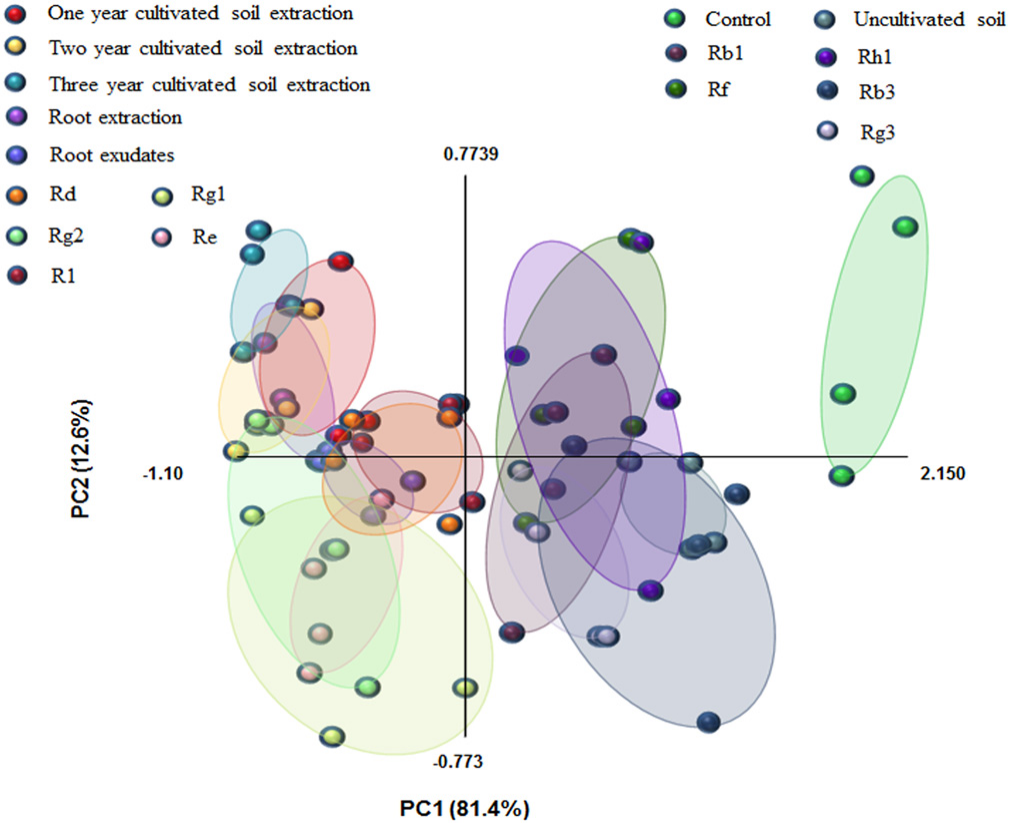
Autotoxicities of the soil extracts, root extracts, root exudates, and ginsenosides against the seedling germination and growth of Sanqi ginseng according to principal component analysis.

The data from all of the treatments were analyzed using PCA to identify the primary factors causing the autotoxicity of Sanqi ginseng. Based on the PCA analysis, we observed that the control and treatments formed three different groups (Fig. 4). The first principal component (81.4%) revealed that several treatments (including extracts from the soils that were continuously cultivated for one to three years, crude ginsenosides, root exudates, and the R_1_, Rg_1_, Re, Rg_2_, and Rd ginsenosides) were clustered into one group and that other treatments (the uncultivated soil and the Rb_1_, Rh_1_, Rf, Rb_3_, and Rg_3_ ginsenosides) without significant autotoxicity were clustered into another group. The control was separate from all of the treatments. These results clearly indicated that the ginsenosides (R_1_, Rg_1_, Re, Rg_2_, and Rd) present in the roots, cultivated soils and root exudates exerted important autotoxic effects on the seed germination and seedling growth at a concentration of 1.0 μg mL^-1^.

**Fig 5:**
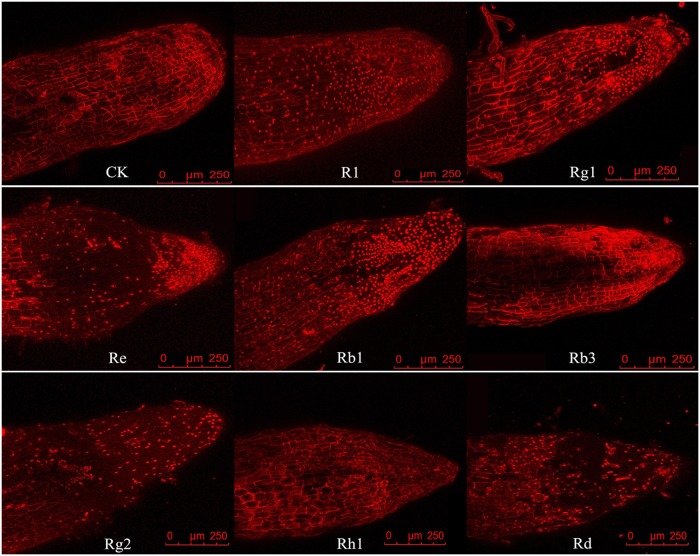
Cell deaths in the Sanqi ginseng root tips after treatment with ginsenosides. Roots were treated with R_1_, Rg_1_, Re, Rb_1_, Rb_3_, Rg_2_, Rh_1_, and Rd at a concentration of 1.0 μg mL^-1^ for 24 h and then stained with PI (5.0 μg mL^-1^). Cell nuclei that stained with PI indicated dead cells (red).

**Fig 6:**
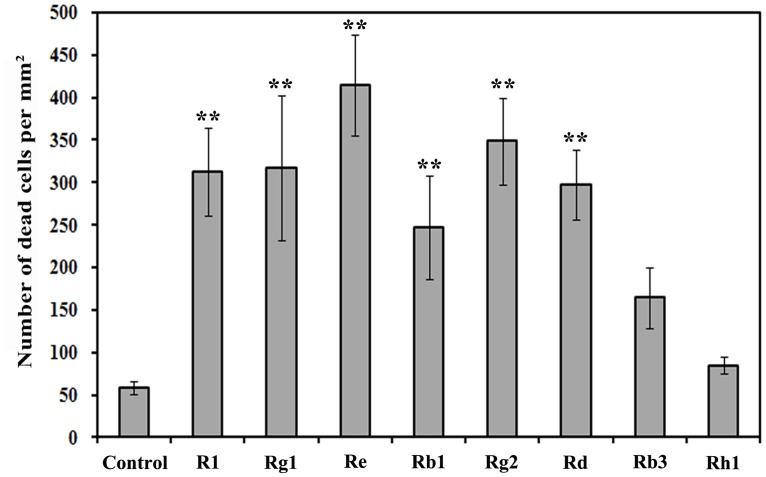
Dead cells in the apical and subapical roots of the Sanqi ginseng after treatment with ginsenosides. Asterisks indicate statistically significant difference of treatment compared with control. *, *p* < 0.05; **, *p* < 0.01, LSD test.

To further test the autotoxicities of ginsenosides in root cells, we performed a PI staining analysis to determine the cell viabilities of the treated and untreated roots. Although PI is excluded by viable cells, it can penetrate the cell membranes of dying or dead cells and can intercalate double-stranded nucleic acids [35]. As shown in Fig. 5, the apical and subapical roots exhibited either a sporadic pattern of PI-stained cells or no stained cells at all. However, the number of PI-positive cells and the staining intensity significantly increased near the apical and subapical root cells after treating with R_1_, Rg_1_, Re, Rb_1_, Rg_2_ and Rd at a concentration of 1.0 μg mL^-1^ for 24 h (Fig. 5). The staining analysis showed that the number of dead cells distributed in the apical and subapical roots was significantly higher than that of the control treatment after treating with the R_1_, Rg_1_, Re, Rb_1_, Rg_2_ and Rd ginsenosides for 24 h (Fig. 6). However, the Rb_3_ and Rh_1_ ginsenosides did not significantly influence the vigor of the root cells relative to those of the control treatment (Fig. 6).

## Discussion

Our results demonstrated that autotoxicity is a factor in the replant failure of Sanqi ginseng. Under greenhouse conditions, we confirmed that the severity of replant failure significantly increased with the number of continuous cultivation years. Particularly, no seedling could survive for more than six months in the soil that was consecutively cultured for three years. Many factors are believed to contribute to crop replant failure, including the deterioration of soil physicochemical characteristics, nutrient imbalance, soil-borne diseases, and autotoxicity [4–5]. During the last three decades, a wide variety of soil-borne pathogens have been identified and linked to the replanting failure of Sanqi ginseng [6–9]. In this study, we observed that autotoxicity can cause Sanqi ginseng replant failure in continuously cultivated soils. In the hydroponic experiment, activated carbon improved the survival of the seedlings grown in the Sanqi ginseng root exudates. Furthermore, the extracts from the roots and the soils in which Sanqi ginseng were consecutively cultivated significantly inhibited seed germination and seedling growth. These data demonstrated that the occurrence of potential autotoxic factors in the soil affects the growth of Sanqi ginseng. Recently, the autotoxicities of other *Panax* species, including *P*. *ginseng* and *P*. *quinquefolium*, have also been identified [18–23].

Several groups of chemicals have been implicated in autotoxicity, including terpenoids, phenolics, steroids, alkaloids, and cyanogenic glycosides [38]. Previous studies indicated that alfalfa root terpenoids (saponin) showed allelopathic activity against the growth and development of some weeds [39]. Ginseng saponin (ginsenoside) is a major effective component that is produced by Sanqi ginseng plants [26]. Several studies have indicated that medicinal plant allelochemicals showed homology to this effective component [40–41]. Thus, we speculated that the ginsenosides were potential autotoxic compounds of Sanqi ginseng. Our greenhouse experiment confirmed that adding root powder to the soil significantly inhibited and suppressed seedling emergence and seedling growth. Furthermore, the crude ginsenosides that were extracted from the roots showed obvious autotoxicity, which demonstrated that ginsenosides are potential autotoxins.

Ginsenosides can accumulate in the rhizosphere soil of Sanqi ginseng from the decomposition of plant residues and from root exudates. In this study, we confirmed that eight major ginsenosides, namely R_1_, Rg_1_, Re, Rf, Rb_1_, Rb_3_, Rg_2_, Rh_1_, and Rd, could be extracted from Sanqi ginseng roots (based on HPLC-MS). This finding is consistent with previous findings [26]. More importantly, we discovered all of these ginsenosides except Rh_1_ in the plow layer of the soil from the Sanqi ginseng fields. In addition, Nicol et al. (2003) confirmed the presence of the Rb_1_, Rb_2_, Rc, Rd, Re, F_11_, and Rg_1_ ginsenosides in the soil extracts of American ginseng using HPLC-MS [28]. These data demonstrated that the ginsenosides detected in the soils of the cultivated Sanqi ginseng fields can be traced to the roots of Sanqi ginseng. In addition, our field studies indicated large amounts of fibrous root waste in the soil after harvest. Nicol et al. (2003) confirmed that the ginsenosides (Rb_1_, Rb_2_, Rc, Rd, Re and Rg_1_) that were recovered from the root-associated soil were present in the root exudates [28]. In our assay, we only identified the Rg_1_, Re, Rg_2_, and Rd ginsenosides in the root exudates using HPLC-MS data. This finding could be explained by the small concentrations of these compounds in the root exudates or by the limitations of our detection method. Overall, it is likely that the ginsenosides found in the soils were partly derived from the root exudates or from root decomposition.

Some ginsenosides showed autotoxicity against seed germination and seedling growth at rhizosphere-relevant concentrations. In this study, we found that the content of a single ginsenoside varied from 0.12 to 1.71 μg g^-1^ (dry weight) in the soil that was continuously cultivated for one to three years. Thus, we selected a ginsenoside concentration of 1.0 μg/mL to test the autotoxicity. These results indicated that the R_1_, Rg_1_, Re, Rg_2_, and Rd ginsenosides exerted autotoxic effects on seed germination and root cell vigor at a concentration of 1.0 μg/mL. The PCA analysis indicated that the autotoxicity of the R_1_, Rg_1_, Re, Rg_2_ and Rd ginsenosides partly overlapped with the autotoxicity shown in the extracts from the consecutively cultivated soil and fibrous roots. However, we found that the inhibitory effects of each ginsenoside were not as potent as the extracts from the soils and roots when tested separately. This finding may be explained by the coexistence of these ginsenosides in the soils, which will cause a stronger autotoxic effect when the ginsenosides function synergistically in natural settings. Similar results have also been reported for other plant species [40, 42]. In addition, other autotoxic compounds could be present in the soil. Some investigators have noted that phenolic compounds can inhibit seedling germination and growth in American ginseng [20]. Thus, further studies are required to determine whether other compounds are present in the soil and whether they are linked to the autotoxicity in Sanqi ginseng.

Ginsenosides are primary components of ginseng and play a multi-purpose ecological role. Like other saponins, ginsenosides are fungitoxic in plants and act as a host for chemical defenses at high concentrations [43]. In the rhizosphere, ginsenosides not only exert autotoxic effects on Sanqi ginseng plants, resulting in poor defense and growth, but also stimulate the growth of soil-borne pathogens. Previous reports have shown that ginsenosides can stimulate the growth of soil-borne *P*. *quinquefolium* and *P*. *notoginseng* pathogens, such as *Cylindrocarpon destructans*, *Fusarium solani*, *Phytophthora cactorum*, and *Pythium irregulare* [27–30]. If the autotoxicity of ginsenosides and the infection of soil-borne pathogens act synergistically in the rhizosphere, a potentially stronger replant failure effect is expected. Some previous studies have indicated that the interactions between *Fusarium oxysporum* and autotoxic cinnamic acid may enhance soil sickness [44–45]. Thus, additional studies are needed to evaluate the interactions between autotoxicity and the other factors involved in replant failure, such as soil-borne pathogens, the deterioration of soil physicochemical properties, and the imbalance of the soil microbial community. These studies could be used to understand the mechanisms of replant failure in Sanqi ginseng.

## Conclusions

Autotoxicity is a cause of replant failure in Sanqi ginseng. Some ginsenosides can accumulate in soils through root exudates or root decomposition and show autotoxicity against seed germination and seedling growth at soil-relevant concentrations. These results provide new insights into the allelopathic mechanisms involved in Sanqi ginseng replant failure.

## Supporting Information

S1 Dataset RAW mass spectrometry data for all identified ginsenosides. (ZIP)

S2 Dataset Calibration curve for all identified ginsenosides. (XLSX)
