# Supplementary material for: Autotoxic Ginsenosides in the Rhizosphere Contribute to the Replant Failure of Panax notoginseng
Source: PLoS One. 2015 Feb 19;10(2):e0118555. doi: 10.1371/journal.pone.0118555 (PMC4335038; doi:10.1371/journal.pone.0118555)
Supplement: S1 Dataset — (ZIP) [file pone.0118555.s001.zip › RAW mass spec data/Mass spec data of Rg2 identified in all samples.docx]

1. Rg2 (authentic ginsenoside standard)

1. Rg2 identified in KG-3 sample

1. Rg2 identified in One-CS sample

1. Rg2 identified in Two-CS sample

1. Rg2 identified in Three-CS sample

1. Rg2 identified in Res sample
